# Ozanezumab Dose Selection for Amyotrophic Lateral Sclerosis by Pharmacokinetic-Pharmacodynamic Modelling of Immunohistochemistry Data from Patient Muscle Biopsies

**DOI:** 10.1371/journal.pone.0117355

**Published:** 2015-02-23

**Authors:** Alienor Berges, Jonathan Bullman, Stewart Bates, David Krull, Nicola Williams, Chao Chen

**Affiliations:** 1 Clinical Pharmacology Modelling and Simulation, GlaxoSmithKline, London, United Kingdom; 2 Biopharm Translational Medicine, GlaxoSmithKline, Stevenage, United Kingdom; 3 Safety Assessment, GlaxoSmithKline, Research Triangle Park, North Carolina, United States of America; 4 Clinical Statistics, GlaxoSmithKline, Stevenage, United Kingdom; Northeastern University, UNITED STATES

## Abstract

Amyotrophic Lateral Sclerosis (ALS) is a rare and fatal neurodegenerative disease with a high unmet medical need. In this context, a potential therapy should be brought to patients in the most expeditious way and early exploration of pharmacology is highly beneficial. Ozanezumab, a humanised IgG monoclonal antibody against Nogo-A protein which is an inhibitor of neurite outgrowth, is currently under development for the treatment of ALS and has been recently assessed in 76 patients in a first-in-human study. Inadequate target engagement has been recognised as a major contributing reason for drug trial failures. In this work, we describe the development of a pharmacokinetic-pharmacodynamic (PKPD) model using immunohistochemistry (IHC) data of co-localization of ozanezumab with Nogo-A in skeletal muscle as a surrogate measure of target engagement. The rich plasma concentration data and the sparse IHC data after one or two intravenous doses of ozanezumab were modelled simultaneously using a non-linear mixed-effect approach. The final PKPD model was a two-compartment PK model combined with an effect compartment PD model that accounted for the delay in ozanezumab concentrations to reach the site of action which is skeletal muscle. Diagnostic plots showed a satisfactory fit of both PK and IHC data. The model was used as a simulation tool to design a dose regimen for sustained drug-target co-localization in a phase II study.

## Introduction

Amyotrophic Lateral Sclerosis (ALS) is a rare neurodegenerative disorder characterised by progressive loss of motor neurons throughout the central nervous system. The disorder is associated with severe neurologic morbidity including widespread skeletal muscle weakness and atrophy which involves respiratory muscles. Approximately 85% of patients die in the first five years following onset [1].

ALS is classified as an orphan disease, i.e. a rare medical condition with a lack of safe and efficacious drugs that, in this case, slow or reverse the decline of function and significantly prolong life. Riluzole, the only approved drug for ALS affecting the disease course, has a modest effect of prolonging survival by about 2 to 3 months but only a marginal effect on function [2] [3] [4] [5]. Developing new drugs for ALS is challenging, mainly because of its unknown pathogenesis and its heterogeneity in terms of clinical and genetic features [6] [7]. Since the approval of riluzole, the field has seen many failed trials. In this context, early exploration of any indication of drug pharmacology would be highly beneficial.

Nogo-A is an inhibitor of neurite outgrowth. It is present in oligodendrocytes and CNS myelin membranes [8] [9]. Nogo-A has been demonstrated to be over-expressed in the skeletal muscle of ALS subjects, and has been proposed as both an early diagnostic biomarker of ALS, and a surrogate of disease severity [10] [11] [12].

Ozanezumab, a humanized immunoglobulin sub-class1 (IgG1)-type monoclonal antibody (mAb) against Nogo-A, is being investigated for the treatment of ALS. The safety, tolerability and pharmacokinetics (PK) of single and repeat intravenous (IV) ozanezumab doses in ALS patients have been evaluated in a first-in-human (FiH) study [13]. In the absence of a direct measure of target binding or target pharmacology in muscle, biopsy samples were examined by immunohistochemistry (IHC) and quantified using laser scanning cytometry (LSC) to support the analysis. The resulting IHC measures included i) the percentage of muscle fibre membrane expressing Nogo-A, ii) the percentage of muscle fibre membrane with co-localized ozanezumab and iii) the percentage of muscle fibre membrane Nogo-A co-localized with ozanezumab.

Described in this paper is an exposure-response analysis that was conducted to support dose selection for subsequent trials. The objectives were: i) to develop a pharmacokinetic-pharmacodynamic (PKPD) model using those IHC data to describe the Nogo-A-ozanezumab co-localization as a surrogate biomarker for drug-target binding; and ii) to simulate the co-localization for a range of not-yet-tested dosing regimens to help the design of future clinical trials. The model-based analysis allowed the integration of multiple types of observational data to provide pharmacological insight [14] [15].

## Methods

The PKPD model was developed using the data generated in the randomised, placebo-controlled, double-blind, two-part, dose-escalation FiH study for IV ozanezumab in subjects with ALS (Trial Registration: ClinicalTrials.gov NCT00875446) [13]. The protocol, protocol amendments, and informed consent of that trial were approved by a national, regional or investigational center ethics committee or an institutional review board (IRB), at each of the participating sites. This study was conducted in accordance with Good Clinical Practice and the guiding principles of the Declaration of Helsinki, and all subjects provided written informed consent. In Part 1, subjects in five cohorts received a single dose of 0.01 to 15 mg/kg, or placebo. In Part 2, subjects in three cohorts received two doses of 0.5 to 15 mg/kg, or placebo, approximately 4 weeks apart (Table 1). This study was described by Meininger *et al* [13]. Details about study design, sample collection and sample analysis were provided in the supporting information of that paper. Methods for PK and PD sample collection, processing and analysis are summarised below.

**Table 1:** Design of the first-in-human study.

Cohort	n placebo	n ozanezumab	Pre-dose 1biopsy	Dose 1(mg/kg)	Post-dose 1biopsy	Dose 2(mg/kg)	Post-dose 2 biopsy
Part 1: A single intravenous dose							
**1**	2	6		0.01			
**2**	2	6		0.10			
**3**	2	6	✔^1^	1.00	✔^1, 3^		
**4**	2	6		5.00			
**5**	2	6	✔^2^	15.00	✔^2, 3^		
Part 2: Two intravenous doses four weeks apart							
**6**	3	9	✔^2^	0.50		0.50	✔^2, 3^
**7**	3	9	✔	2.50		2.50	✔
**8**	3	9	✔	15	✔^4^	15.00	

^1^ Biopsy with freeze artifact making them unsuitable for the IHC analysis

^2^ Biopsy analysed by the pilot LSC assay hence unsuitable for inclusion in PKPD analysis

^3^ Biopsy collected at day 22–28 post dose

^4^ Biopsy collected at either day 1, day 8 or day 22–24 post dose

### PK data collection and analysis

Blood samples to characterise the PK of ozanezumab in plasma were collected at multiple times in both parts of the study. In Part 1, blood PK samples were collected before dosing; at 1, 10 and 24 hours after dosing; and at 2, 4, 6, 8 and 12 weeks after dosing. In Part 2, blood PK samples were collected before the first dose; at 1, 10 and 24 hours after the first dose; and at 2, 4, 8, 10, 12 and 16 weeks after the first dose. Additional blood PK samples were collected before and at 1 and 6 hours after the second dose, which was given 4 weeks after the first dose. Two deltoid muscle biopsies were taken in each subject, where possible from the same muscle: one pre-dose and one post dosing at various times across cohorts (Table 1). Muscle lysates were generated by homogenisation of tissue in lysis buffer (55mM Tris-HCl, 165mM NaCl, 10% Triton, 1mM EDTA, Halt protease and phosphatase inhibitor cocktail) and protein concentrations estimated using bicinchoninic acid (BCA) analysis. Plasma and muscle lysates were analysed for ozanezumab using a validated enzyme-linked immunosorbent assay.

### IHC preparation and LSC detection

Frozen muscle biopsies were examined by IHC and LSC for the level of proteins including Nogo-A and ozanezumab. Frozen sections were stained with antibodies against Nogo-A, a non-neutralising anti-idiotypic antibody against ozanezumab, and an antibody against gamma sarcoglycan, and then detected using a combination of direct and indirect fluorescent staining using the Intellipath automated IHC instrument (Biocare Medical). An iCyte LSC cytometer (CompuCyte Corp) was used to localize and quantify the fluorescence staining associated with each antibody. After initial optimization of the methodology using samples from early cohorts, samples from Cohorts 7 and 8 were analysed as a single batch (using triplicate sections from each biopsy to understand biological variability) and were used in PKPD analysis. As the intracellular expressed Nogo-A is not available for binding to ozanezumab, gamma sarcoglycan staining was used as a marker of muscle plasma membrane and allowed an estimation of membrane-associated Nogo-A, ozanezumab and their co-localization. The resulting endpoints for PKPD model development were: percentage of the membrane expressing Nogo-A, percentage of the membrane with co-localized ozanezumab, and percentage of the membrane Nogo-A co-localized with ozanezumab.

### PKPD analysis

Exploratory data analyses were undertaken using plasma and muscle ozanezumab concentrations, together with IHC data to assess the data suitability, identify potential outliers, appreciate variability of the assays and inform model development. The LSC measures from the first batch were excluded from the PKPD analysis as the original gating strategy and cytometer settings in the pilot methodology were not suitable for pooled analysis with the data obtained with the optimized final method. In addition, freeze artifacts made the muscle samples from cohort 3 unsuitable for the IHC analysis (Table 1).

The modelling was conducted in software NONMEM (version7.1.2, ICON Solutions) [16] and PsN (Perl Speaks NONMEM, version 3.2.4) [17] using a non-linear mixed-effect approach [18]. The standard method of first-order conditional estimation with interaction was used to estimate model parameters.

PK and PD parameters of the model were estimated simultaneously with the plasma concentrations of ozanezumab in all subjects (including those without biopsy) and the IHC measures of co-localization from subjects with muscles biopsies at all doses including placebo. The other IHC outcomes (i.e. the percentage of the membrane expressing Nogo-A and the percentage of the membrane with co-localized ozanezumab) and the muscle concentrations were used in model exploration, but not included in the final model.

The model performance was judged by convergence status, covariance estimation, parameter estimation precision, likelihood ratio test [19], standard goodness-of-fit plots [20] [21] and concordance of estimated parameters with values found in the literature. Models were evaluated using simulation-based diagnostics such as visual predictive check (VPC), which allowed visual comparison of the model-simulated data with the observed data, in order to detect notable model misspecifications [21]. The VPC was produced using 5th, 50th and 95th percentiles obtained from 300 simulations; and the comparison for PK and IHC measures was stratified by dose regimen.

### Model Simulations

The final model with the estimated population parameter values was implemented using the deSolve package in R software (version 3.0.1) [22]. Median plasma ozanezumab concentration and co-localization were simulated for a range of dosing conditions to support the dosing rationale for future studies. The level of 90% co-location was arbitrarily set as the criterion for required pharmacology.

## Results

### PKPD data

**Fig 1:**
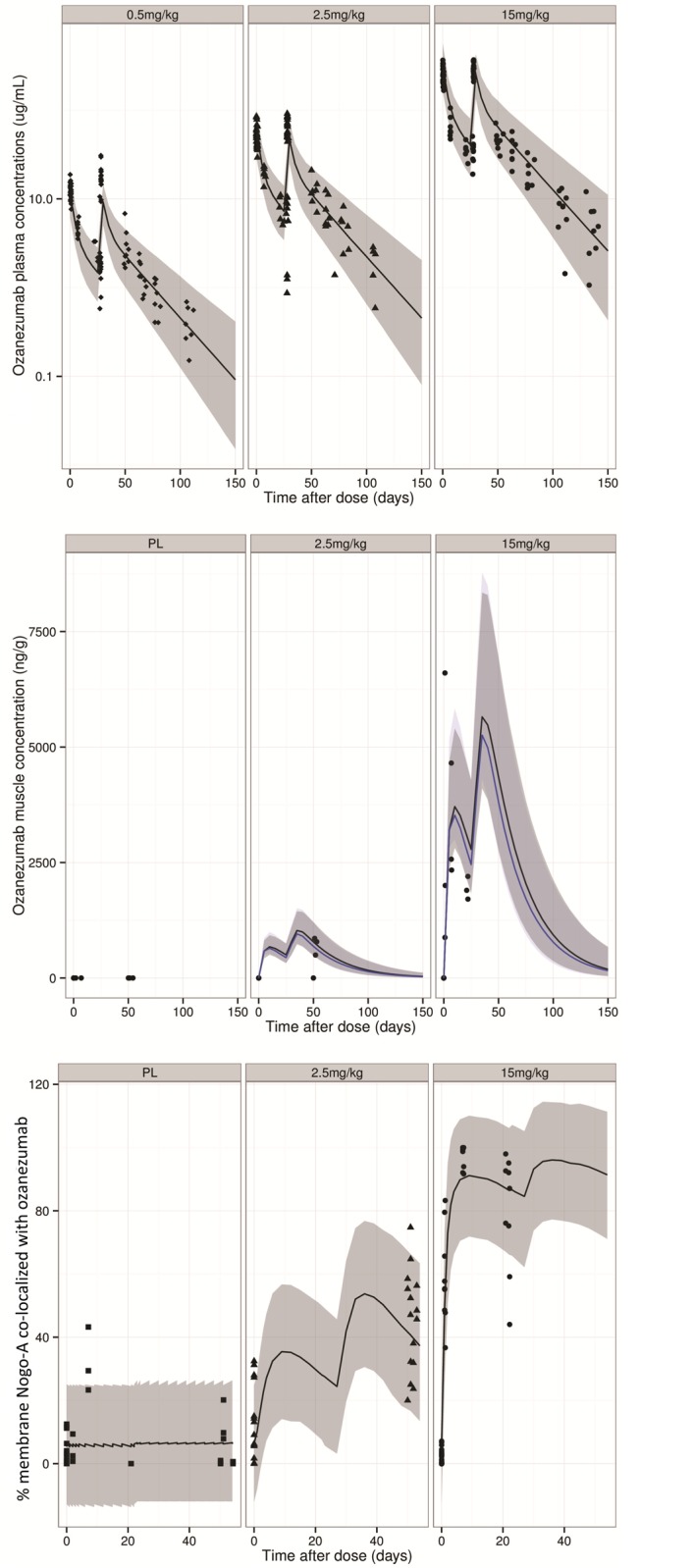
Plasma drug concentration (top), muscle drug concentration (middle) and co-localization of Nogo-A with drug (bottom). In the top and bottom panels: visual predictive check for final model is shown (the points are the observations, the black line is the median of the simulations and the ribbon delimits the 5th and 95th percentiles of the simulations). In the middle panel: points are the observations, black line and dark ribbon represent model-simulated median and 5^th^—95^th^ percentile of effect compartment concentration, blue line and light ribbon represent model-simulated median and 5^th^—95^th^ percentile of peripheral compartment concentration.

**Fig 2:**
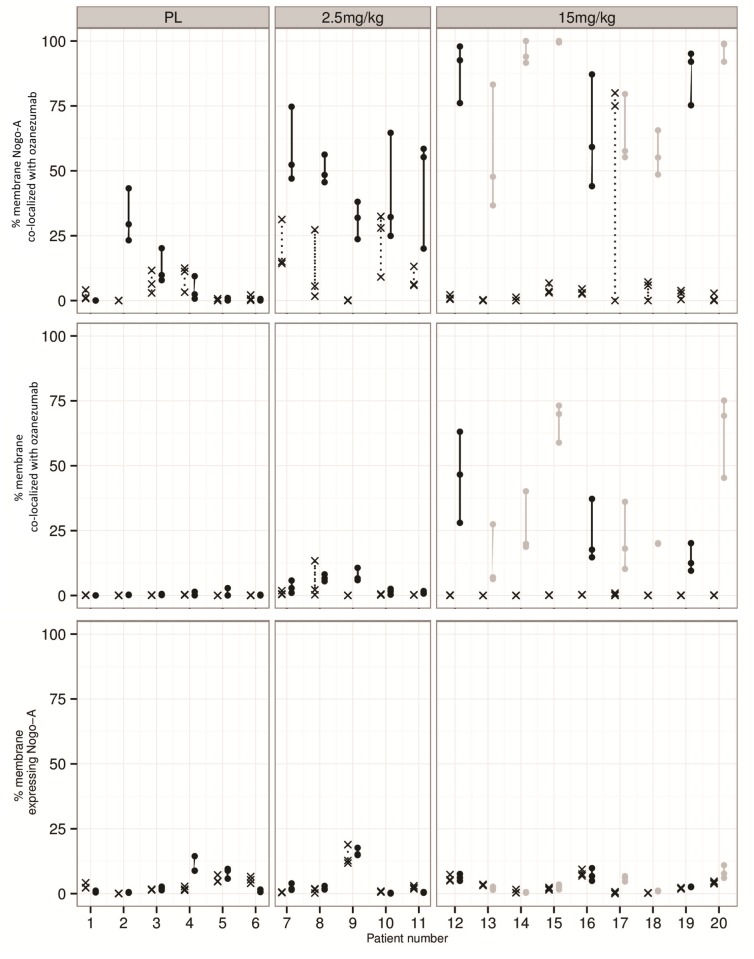
IHC triplicate measures from skeletal muscle biopsies per individual subjects at pre-dose (cross), ≤ 10 days post dose (grey dot), and > 10 days post-dose (black dot).

Plasma ozanezumab concentration-time profiles were available for all subjects in Part 1 and in Part 2. Muscle ozanezumab concentrations were limited to the patients with muscle biopsies. Fig. 1 illustrates the PK profiles from Part 2, which was repeat dosing, both in plasma (upper panel) and in muscles lysates (middle panel). The plasma ozanezumab PK profiles showed a peak concentration proportional to the dose at the end of infusion, followed by a bi-exponential decline with a terminal half-life of approximately 20 days. Muscle ozanezumab concentrations, quantifiable only in a subset of subjects, increased with increasing dose. A similar trend was seen in the detection of ozanezumab in tissue samples by IHC (Fig. 2, bottom panel).

**Fig 3:**
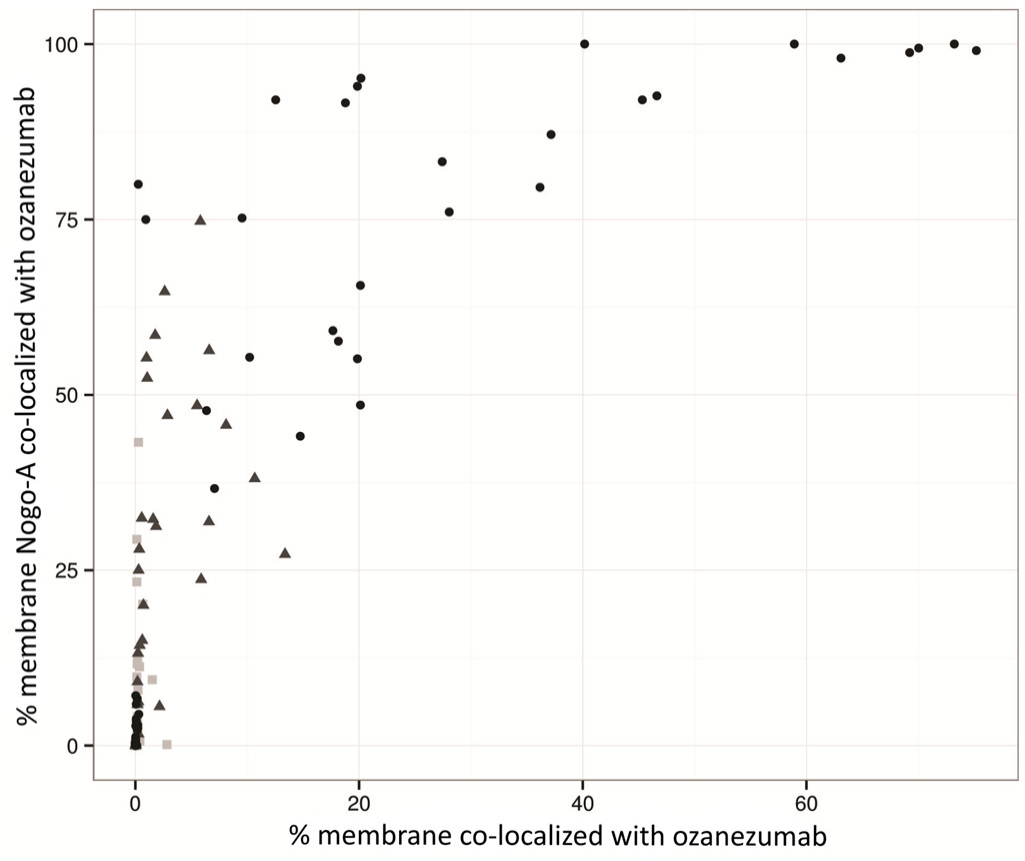
Percentage of membrane Nogo-A co-localized with ozanezumab versus percentage of membrane with co-localized ozanezumab (square for placebo, triangle for 2.5 mg/kg, and dot for 15 mg/kg).

The IHC measures that were used for the PKPD analysis, from cohorts 7 and 8, are shown in Fig. 2. While there was clear variability in the IHC measurements, there was generally good concordance between triplicate measures and trends were readily notable. The percentage of membrane expressing Nogo-A ranged from 0.0% to 17.7%; it remained stable over time and showed no response to drug treatment (bottom panel). Some background staining with the anti-idiotypic antibody against ozanezumab led to apparent measured drug and its co-localization with Nogo-A in pre-dose or placebo samples (middle and top panels). The biopsy sample at pre-dose from patient 17 in the 15 mg/kg cohort was too small for reliable IHC measurement; the values were removed for the analysis. Overall, both the percentage of the membrane co-localized with ozanezumab and the percentage of the membrane Nogo-A co-localized with ozanezumab increased with dose (Fig. 2) and correlated with each other (Fig. 3).

### PKPD model

The final PKPD model is a two-compartment PK model, with dosing and elimination both in the central compartment, combined with an effect compartment PD model that accounts for the delay in ozanezumab concentrations to reach the site of action from the central compartment [24]. This model is described by equations 1 to 4.

$$\frac{dAc}{dt}=IR+\frac{Q}{Vp}*Ap-\frac{CL}{Vc}*Ac-\frac{Q}{Vc}*Ac$$ Equation 1

$$\frac{dAp}{dt}=\frac{Q}{Vc}*Ac-\frac{Q}{Vp}*Ap$$ Equation 2

$$\frac{dCe}{dt}=keo*\frac{Ac}{Vc}-keo*Ce$$ Equation 3

$$E=E0+(Emax-E0)*\frac{Ce^{\gamma }}{EC50^{\gamma }+Ce^{\gamma }}$$ Equation 4

For Equations 1–4, Ac, Ap, Ce and E are drug amount in central compartment (mg), drug amount in peripheral compartment (mg), drug concentration at effect site (ug/mL) and proportion of membrane Nogo-A that is co-localized with the drug, respectively. Variable IR is drug dosing rate (ug/h). Parameters Q, Vp, CL and Vc are inter-compartment clearance (mL/h), peripheral compartment volume (mL), elimination clearance (mL/h), and central compartment volume (mL), respectively. Parameter keo (1/h) determines the delay of the concentration from central compartment to the effect site [23]. While E0 and Emax are baseline level and maximal level of the proportion of membrane Nogo-A that is co-localized with the drug, EC50 (ug/mL) is the drug concentration causing 50% of the drug effect and *γ* governs the sigmoidity of the Emax model [24]. The model was fitted to observations of plasma concentration of ozanezumab (Ac/Vc) and proportion of membrane Nogo-A that is co-localized with the drug (E).

Given the limited biopsy data available per subject, between-subject variance was applied only to the PK parameters CL and Vc. A proportional residual error was applied for the PK measurements; and an additive residual error was applied for the PD measurements. An additional error term was used to account for the variability amongst the three replicate PD measurements, implemented via the L2 data item in NONMEM [25]. The final model parameter estimates are listed in Table 2. All parameters were estimated with reasonable precision (highest relative standard error (RSE) was 26%), except for the residual error that was not replicate-specific (RSE = 56%). The between-subject variance and the residual error for PK were moderate (20–25%). The residual errors for the PD measures were relatively high (SD of 6.16% and 9.47%), reflecting the nature of the biopsy and the IHC method.

**Table 2:** Summary of ozanezumab PK/PD model parameters.

Parameter	Parameter definition	Value	RSE (%)
CL (mL/h)	Elimination clearance	11.7	4.2
V_C_ (mL)	Volume of the central compartment	3310	4.3
V_P_ (mL)	Volume of the peripheral compartment	3650	5.0
Q (mL/h)	Inter-compartmental clearance	14.6	8.6
E_max_ (%)	Maximal proportion of the membrane Nogo-A that is co-localized with ozanezumab	100 (Fixed)	
E_0_ (%)	Proportion of the membrane Nogo-A that is co-localized with ozanezumab in absence of the drug	6.04	26
EC_50_ (μg/mL)	Ozanezumab concentration causing 50% of the maximal drug effect	24.9	10
γ	Sigmoidity of the E_max_ model	1.94	14
k_e0_ (1/h)	Rate constant for the concentration delay between central compartment and the effect site	0.00359	14
Omega CL	Variance of between-subject variability of CL (CV)	0.063 (25%)	21
Omega V_C_	Variance of between-subject variability of V_C_ (CV)	0.0401 (20%)	20
CV PK (%)	Coefficient of variation of PK residual	25.4	5.0
Sigma PD	Variance for PD residuals (SD)	38.0 (6.16)	56
Sigma PDr	Variance among PD replicates (SD)	89.7 (9.47)	26

RSE: Parameter Estimation Standard Error/Parameter Estimate

### Model evaluation

The simulation-based diagnostic plots showed that the model adequately described the temporal pattern of both the PK and PD observations (Fig. 1, top and bottom panels). Furthermore, comparing the 90% model prediction interval to the distribution of the observations suggested that model adequately captured the variability in both PK and PD data.

### Simulations

**Fig 4:**
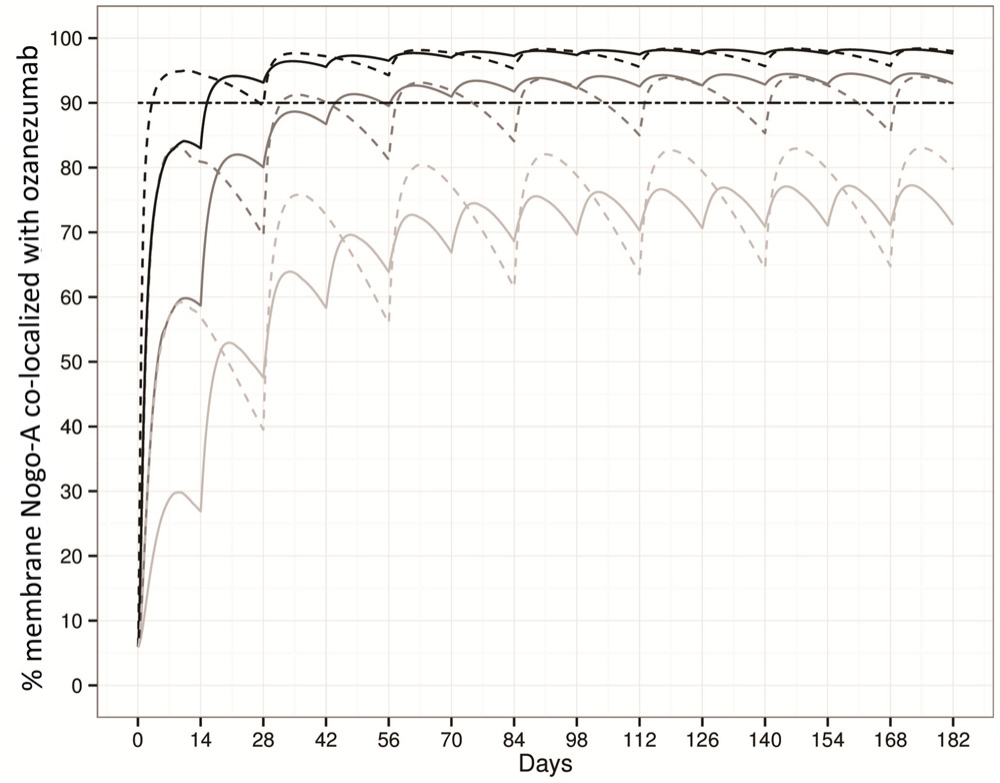
Model simulations of percentages of membrane Nogo-A co-localized with ozanezumab following one-hour infusion at 5, 10 and 20 mg/kg/h every 28 days (dashed lines); and at 2.5, 5 and 10 mg/kg/h every 14 days (solid lines).

The PKPD model was used to help understand the impact of dose and dosing frequency on the extent and sustainability of PD response in term of Nogo-A and ozanezumab co-localization on muscle membrane. For example, the model-simulated time course of co-localization is shown in Fig. 4 for the same total monthly doses of 5, 10 and 20mg/kg, administered as one-hour intravenous infusions, given either as full doses every four weeks or as half doses every two weeks.

As expected, higher doses were predicted to produce greater co-localization regardless of regimen; dose-splitting was predicted to lead to less response fluctuation over time. At 5mg/kg or 20mg/kg per four weeks, both regimens would produce consistently over time a lower or higher than the arbitrary level of 90% co-localization. At the 5mg/kg dose, both regimens would reach overall similar level of co-localization, but more frequent dosing would lead to less fluctuation. The lower fluctuation at higher doses reflects the plateau at high concentrations of the concentration-response relationship that is captured by the Emax model. With the same total dose of 10mg/kg per four weeks, the co-localization is always above 90% with the split-dose regimen, but fluctuates over time around 90% with the alternative regimen.

## Discussion

In this work, we developed a PKPD model using IHC data from muscle biopsies collected in a FiH study to predict the co-localization of membrane Nogo-A and ozanezumab as a function of ozanezumab dosing regimen.

Adequate target binding by the drug molecule is a prerequisite for producing a clinically relevant level of pharmacology. A dose that is intended to provide efficacy in a patient trial should be supported by evidence that it is associated with sufficient level of target binding in a sustained fashion. In the absence of an in vivo assay that can be used to quantify the extent of Nogo-A binding by ozanezumab, we used the percentage of membrane Nogo-A co-localized with ozanezumab as a surrogate for the target binding. The LSC technology represents a novel approach to tissue cytometry that provides quantitative measurements simultaneously with image collection. Its preclinical applications to support drug discovery in target screening, biomarker identification and routine histopathology have already been described in the literature [26] [27]. To the authors’ knowledge, this is the first instance of applying LSC-quantified co-localization data in clinical studies. Such method may have broader utility as a biomarker of pharmacology in other situations.

The PK of ozanezumab was adequately described by a two-compartment linear model, typical for a human IgG-type molecule without clear evidence of target-mediated disposition which has sometimes been observed for drugs of this class [28]. This was also consistent with the previously reported dose-proportionality of maximal concentration and dose-independency of clearance for this drug [13]. During model development, we explored the inclusion of muscle drug concentration and the percentage of membrane with co-localized ozanezumab as observations in addition to plasma drug concentration. The addition of these data separately or together, to drive drug-target co-localization in the muscle, failed to improve model fit yet reduced model stability. Hence formal model building only included the plasma concentrations, with the parameter ke0 to account for the plasma-to-muscle delay in drug appearance. Nonetheless, the ke0 of the final model was estimated to be 0.0036/h, similar to the peripheral-to-central transport rate constant which can be derived as Q/Vp = 0.0040/h (see Table 2). This similarity suggests that the time course of drug concentration in the effect compartment is consistent with that in the peripheral PK compartment, as shown by the near identical model-simulated data envelopes for these two compartments (Fig. 1, middle panel). This suggestion is supported by the observation that, despite the small number of samples with measured muscle concentrations, the time course of muscle concentration levels was consistent with the time course of the model-simulated peripheral compartment data envelope (Fig. 1, middle panel). In addition, the delay between the plasma PK and the muscle co-localization as characterised by ke0 in the model could be explained by the tissue distribution of ozanezumab. This is in line with the typical distribution of a large and highly polar molecule like an mAb, which is much slower than that of most small molecules and mainly driven by extravasation via the microporous endothelial barrier [29].

A minimal physiologically-based pharmacokinetic model (mPBPK) approach was recently proposed for PK modelling of mAbs [30]. While conventional full PBPK models require drug concentrations from many tissues, the mPBPK approach allows inference of tissue concentrations when only plasma drug concentrations are available under certain mAb-specific assumptions. As an exploratory analysis, we fitted the plasma concentration of ozanezumab to an mPBPK model, fixing the available fraction of interstitial fluids (ISF) for mAb distribution to 0.8 and lymphatic capillary reflection coefficient to 0.2 [30]. Clearance from plasma was estimated to be 0.0126 L/h (RSE 5%), not dissimilar to the estimate for the compartmental PK model (Table 2). Vascular reflection coefficients for ISF in tissues with continuous and discontinuous capillaries were estimated to be 0.826 (RSE 11%) and 0.497 (RSE 12%), respectively. These parameter estimates were consistent with those reported for other mAbs in Cao et al [30].

The lack of a direct measure of drug-target binding is a clear knowledge gap in the development program of this compound. The Emax PKPD model shows that the amount of the target that is co-localized with the drug is saturable when the drug concentration is high. This observation lends support to using the co-localization as a surrogate for drug-target binding.

The PKPD model that was built with limited data collected in a short-term study was used to design a dose regimen, by simulation, for sustained drug-target co-localization in a longer phase II study (Trial Registration: ClinicalTrials.gov NCT01753076). Whether the achieved co-localization would lead to measurable pharmacology (such as maintenance of muscle function) or the ultimate clinical efficacy (such as change in the rate of decline in ALSFR-S or improved mortality measures) remains to be tested.
